# Methods for Capturing and Quantifying Contact Events in Collision Sports

**DOI:** 10.3390/sports13040102

**Published:** 2025-03-27

**Authors:** Craig Bolger, Jocelyn Mara, Byron Field, David B. Pyne, Andrew J. McKune

**Affiliations:** 1 Faculty of Health, Research Institute for Sport and Exercise, University of Canberra, Kirinari Street, Bruce, Canberra, ACT 2617, Australia; craig.bolger@canberra.edu.au (C.B.); jocelyn.mara@canberra.edu.au (J.M.); david.pyne@canberra.edu.au (D.B.P.); 2 ACT Brumbies Rugby, University of Canberra, Building 29, University Drive, Bruce, Canberra, ACT 2617, Australia; 3 Rugby Australia, ARU Building, Moore Park Rd & Driver Ave, Paddington, NSW 2021, Australia; byron.field@rugby.com.au; 4 School of Health Sciences, Biokinetics, Exercise and Leisure Sciences, University of KwaZulu-Natal, Durban 4041, KZN, South Africa

**Keywords:** systems, head protective devices, video recording, algorithms, rugby

## Abstract

Technological advancements have led to widespread use of wearable devices that capture external performance metrics in team sports. Tracking systems including global positioning system (GPS) technology with inbuilt microelectromechanical systems (MEMS), instrumented mouthguards (iMGs), and video analysis provide valuable insights into the contact demands of collision sports. In collision sports, successfully “winning the contact” is positively associated with better individual and team performance, but it also comes with a high risk of injury, posing a concern for player welfare. Understanding the frequency and intensity of these contact events is important in order for coaches and practitioners to adequately prepare players for competition and can simultaneously reduce the burden on athletes. Different methods have been developed for detecting contact events, although limitations of the current methods include validity and reliability issues, varying thresholds, algorithm inconsistencies, and a lack of code- and sex-specific algorithms. In this review, we evaluate common methods for capturing contact events in team collision sports and detail a new method for assessing contact intensity through notational analysis, offering a potential alternative for capturing contact events that are currently challenging to detect through microtechnology alone.

## 1. Introduction

Technological advancements have enabled the detection and application of metrics related to physical performance, physiological status, biochemical composition, and mental alertness in athletes. These innovations can help reduce injury risks and enhance physical performance, enabling coaching, medical, and performance staff to create, evaluate, and refine athlete-centered protocols and treatment plans [1,2]. A key application of technology in sport is the capture and analysis of the demands of the competition, which can play a pivotal role in refining training sessions to replicate or exceed actual match-play conditions, thus promoting athletes’ adaptation and improving their overall performance during competition. Sporting demands are generally classified into external and internal categories. The term “external” refers to the mechanical or locomotive stress placed on an athlete during exercise (e.g., distance travelled, number of jumps performed, or the number of tackles performed in rugby) [3]. “Internal” demands refer to the physiological and psychological stress (e.g., heart rate [HR], rating of perceived exertion [RPE]) experienced by the athlete in response to the external demands [3].

In team sports, external demands are most commonly measured using wearable tracking systems like global positioning (GPS) or local positioning (LPS), which generate large volumes of data that provide valuable insights into the physical demands of match-play. These data help coaches and performance staff to better understand the specific demands players face during match-play [4,5,6,7,8]. When combined with other technologies, such as a heart rate monitor and embedded inertial sensors, these tracking systems play a crucial role in developing a comprehensive understanding of match-play [9]. The information that they provide assists in planning, evaluating, and refining training plans that align with both physical and tactical demands, aiming to improve athletic performance and reduce injury risk.

Under the American Academy of Paediatrics (AAP) framework, collision sports are characterized by the involvement of deliberate, forceful contacts as a defining element of the sport (e.g., rugby union, rugby league, American football, ice hockey, lacrosse). This contrasts with “contact” sports (e.g., soccer, basketball), where participants are exposed to contact events that are not the sport’s central feature and usually happen with less force [10]. In collision sports such as rugby union and rugby league, contact events are a crucial part of the game. In rugby union, the tackle, ruck, and scrum are widely recognized as the most common physical interactions. The outcome of a team’s performance often hinges on their collective ability to face and endure these events in order to secure victory [11,12,13]. Various devices have been developed to capture the frequency and intensity of these physical contact events. Recently, instrumented mouthguards (iMGs) incorporating gyroscopes and accelerometers have become increasingly popular in collision sports, given their ability to capture head acceleration events (HAEs) resulting from direct head impacts, or whiplash caused by contact to other parts of the body [14,15,16]. Other monitoring methods include GPS and LPS devices equipped with accelerometers, gyroscopes, and magnetometers, collectively known as micro-electromechanical systems, or MEMS [17,18], instrumented helmets (e.g., GForceTracker™) [19], headbands (e.g., SIM-G™) [20], and skin patches (e.g., xPatch™) [21]. Contact events captured by these devices are typically detected when changes in position and pre-defined g-force thresholds surpass specified limits [22]. Despite the wide availability of impact detection sensors, recent validation studies have raised concerns about their practical clinical utility to accurately capture head impact events [23,24]. For example, helmet-based systems are limited in their application across different sports, and studies report that skin-mounted sensors can be uncomfortable or easily dislodged during play [21,24].

At present, there are no universally accepted algorithms or thresholds for quantifying contact events. Given the variations in contact dynamics across collision sports, it may be necessary to adopt sport-specific thresholds. These algorithms should be tailored to the specific sport, the competitive level, and the participants’ sex in order to enhance their accuracy and relevance [25,26,27,28]. However, maintaining consistency within individual sports codes is essential for advancing both practice and research. Currently, researchers and manufacturers have applied different thresholds for detecting contact events, leading to inconsistencies in the reported frequency and intensity of contact events [17,18]. Furthermore, specific contact events, such as rucks and mauls in rugby union, present unique challenges for data capture using microtechnology [28]. For example, an athlete may experience contact within a ruck without substantial changes in body orientation, resulting in the event not being captured.

Given the growing focus on athletes’ safety and performance in collision sports, there remains a critical need to establish accurate and reliable methods for detecting and quantifying contact events in collision sports. Despite advancements in technology, current approaches, including video analysis and wearable sensors, have inherent limitations in their ability to quantify contact involvements. Existing research has yet to comprehensively compare these methods in the context of practical application. Therefore, the current review aimed to detail and evaluate the most commonly used methods for capturing contact events in team collision sports (with reference to rugby union) and to identify the advantages and key limitations associated with each approach. We examined the practical application of wearable devices for detecting contact events relevant to both human performance and player welfare. In this review article, we summarize these findings and propose an alternative method for quantifying the intensity of discrete contact events, for use until the available technology can provide satisfactory sensitivity for the detection of contact events. Finally, recommendations for future research are provided.

## 2. Tracking Systems with Inbuilt Inertial Sensors

### 2.1. Data Capture

Since their introduction in 2001, the use of wearable tracking systems such as GPS and LPS devices to capture athlete workload and match demands has increased exponentially [4]. While initial iterations of tracking devices mainly used satellite systems to evaluate the running demands of different sports, most tracking systems now incorporate inertial sensors such as triaxial accelerometers, magnetometers, or gyroscopes (Table 1), providing researchers and practitioners with a wide range of metrics to assess external demands and the movement profiles of players [4,17,29].

With these advancements, researchers routinely undertake independent validity and reliability studies for each device (and its subsequent updates) upon release from commercial suppliers [4,30]. However, given the imperative for early implementation in sports, tracking devices are frequently used before the validity and reliability of the devices have been established [4,31]. With the integration of inertial sensors, manufacturers are continually developing new metrics, largely independent of any research to validate their use [4]. For example, triaxial accelerometers measure acceleration on three different axes (anterior–posterior, medial–lateral, and vertical) [18,29]. The summation of acceleration on these axes yields a vector magnitude (g-force). These new metrics have been utilized to quantify the frequency and intensity of athletes’ involvement in contact events during match-play [28,32,33]. While this approach offers a more time-efficient method for assessing contact events compared with other methods like video analysis, concerns have been raised regarding the reliability and validity of such metrics, with reported underestimations of 98% and overestimations of 74% [28].

Contact metrics tend to be collected in two ways, either (1) by combining accelerometer, magnetometer, and gyroscopic data to identify changes in a players’ body position while also exceeding a pre-defined g-force threshold, or (2) solely through accelerometer-derived data when the player surpasses a pre-defined g-force threshold. While the first approach offers a more comprehensive and potentially valid assessment of various types of contact, the second method is more prone to generating false positives. Without regard for changes in the device’s orientation, such a metric can accumulate non-contact activities (jumping, shuffling, walking, and changes in trunk orientation). This may lead to misrepresentation of participants’ exposure to contact; specifically, overestimation of the actual count of contact events [33]. For example, average peak impact exposure in rugby union has been reported to range between 4 and 6 impacts per minute, with worst-case scenarios including as many as 15 impacts per minute [34]. Another study reported that elite women’s rugby union players recorded over 400 impact events exceeding 5 g during a single match [35]. These values significantly surpass those reported using other microtechnology and video analysis methods [17,32,36].

### 2.2. Practical Applications

With most professional teams already integrating tracking devices such as GPS or LPS into their routine, their use for capturing contact involvement not only eliminates the need for additional costly inertial sensors but also automates data collection and streamlines the workflow for practitioners. Unlike other devices that may cause discomfort or add to a player’s pre-training routine, GPS or LPS systems are already a standard part of training and match-play, reducing both the burden on players and the probability of data being missed. Moreover, tracking systems with embedded MEMSs enable practitioners to capture contact at the drill level, facilitating training estimates, enhancing contact management for players, and improving the overall design of training programs. In addition to quantifying total contact events, different manufacturers employ distinct algorithms for quantifying contact intensity and total contact volume (frequency x intensity) [33,37]. Relying solely on simple contact counts, which treat all contact events equally, may lead to underestimation of the actual contact volume, particularly when the intensity of contact varies substantially. By incorporating metrics that account for both the frequency and intensity of contact events, a more comprehensive and nuanced understanding of cumulative physical stress can be achieved, facilitating more precise monitoring and management of player contact exposure. By applying general training principles such as periodization and progressive overload, practitioners can design or advise on training programs that prepare athletes for the contact demands of their sport, reducing injury risk and enhancing performance. In addition to technical proficiency, emphasis should be placed on developing athletes’ ability to repeatedly engage in contact events, ultimately fostering ‘collision-fit’ athletes. Future research should explore the optimal dosage and type of contact training required to mitigate injury risk, essentially identifying the ‘sweet spot’ in contact exposure.

### 2.3. Limitations

Despite advancements in tracking systems with embedded MEMS for quantifying contact events, several challenges remain. One persistent issue is the difficulty in accurately capturing all contact events that players are exposed to during games and training sessions. For example, an athlete may be exposed to contact within the ruck or during a tackle break without substantial alterations in their body orientation. As a result, these contact events, which may cause substantial damage to the impacted tissue, are not adequately captured. Issues with quantifying contact events via tracking system-embedded MEMSs also stem from the wide range of thresholds used by researchers to detect contact in rugby, with g-force values ranging from 1 g to 8 g [17]. The threshold that yields the most accurate contact count varies depending on the athlete’s sex [26] and playing position [28], as well as the rugby code [28,38], with lower recall observed in females than males (45% vs. 69%), and lower correlations between tracking system-embedded MEMS and video-coded events in rugby union compared with rugby league (union: r = 0.32–0.80 vs. league: r = 0.96). In rugby league, tackles typically occur at higher speeds and involve fewer players, primarily in one-on-one situations, resulting in more pronounced changes in movement that tracking system-embedded MEMS can readily detect. In contrast, rugby union presents more complex contact scenarios, often involving low-velocity, multi-player engagements, such as rucks, mauls, and scrums, which generate less distinct movement patterns, making their detection more challenging.

While newly developed algorithms from tracking system-embedded MEMSs aimed at differentiating contact events showed promise, these were based on one-on-one tackles, thus limiting their broader application [11]. Further challenges arise when comparing rugby union to rugby sevens, where algorithms designed for detecting scrums are less effective for scrums with only three players, such as are seen in rugby sevens [39]. Moreover, manufacturer-specific filtering algorithms that employ temporal rolling periods to reduce false positives can underestimate total contact events, with some research on senior male rugby union players reporting underestimations of 30% for forwards and 43% for backs [40]. If a ball-carrier is tackled consecutively, first by a primary and then secondary tackler, within the manufacturer’s temporal rolling period, the two instances of contact may be incorrectly classified as a single event. Similarly, a tackler who releases the ball-carrier and immediately competes for possession at the ruck may experience two distinct contact events that the tracking system-embedded MEMS would combine into one, thus leading to underestimation of the actual number of contact events [40].

In-house testing has also revealed substantial variations in data derived from accelerometers housed in different vest sizes and in different types of clothing (vest vs. jersey). To ensure the accuracy of MEMS-derived data, the tracking system device needs to be tightly secured within a pocket that limits the irregular movement of the device. However, this is not always possible in an applied setting, as there is often marked variation in jersey fitting and between the pouches that the units are secured in. Furthermore, in these test settings, female players are often required to wear vests or jerseys that are unisex or designed for male anthropometrics. Consequently, players often have issues with uncomfortable fit around the chest and shoulders, which can result in sub-optimal data capture as well as issues with comfort [41]. The magnitude of under- or overestimation of contact involvement demonstrates that using tracking system-embedded MEMS in isolation provides a substantially different portrayal of the contact demands of match-play in collision sports. Consequently, obtaining valid and reliable data on contact events through the use of tracking system-embedded MEMS alone has proven to be a challenge.

Moreover, within these systems, inertia-based metrics for quantifying contact volume are frequently presented without a clear explanation of their derivation. Bespoke metrics like Collision Loadᵀᴹ from StatSports or PlayerLoadᵀᴹ from Catapult have been used to quantify the total magnitude and frequency of contact events without detailed and transparent descriptions of the underlying algorithms or the filtering methods used [27,33,42]. Some researchers recommend metrics such as PlayerLoad Slowᵀᴹ (a measure of accelerometer load while the player is travelling at velocities lower than 2.2 m/s) to indicate contact load [43]; however, other researchers have cautioned its use [3]. Unlike Collision Loadᵀᴹ, which accounts for the magnitude and duration of all contact events detected according to changes in the axis orientation and linear acceleration greater than 8 g-force [33], PlayerLoadᵀᴹ uses simply the sum of accelerometric data collected from all three planes (anteroposterior, mediolateral, and craniocaudal) of motion [3]. Table 2 summarizes the main advantages and disadvantages of these methods.

## 3. Head Impact Telemetry Devices

### 3.1. Data Capture

Athletes participating in collision sports frequently encounter both concussive and non-concussive HAEs [16]. Exposure to these events may be linked to immediate and prolonged neurocognitive changes. As a result, sports governing bodies and researchers have been interested in understanding and quantifying these events in order to reduce the frequency and severity of their occurrence in training and competition [44]. Wearable kinematic sensors, known as head impact telemetry devices, can provide an objective measure of the frequency and intensity of head acceleration events [15]. Currently, a wide variety of head impact devices are commercially available, including instrumented helmets, headbands, mouthguards, and skin patches [24]. These devices typically integrate inertial measurement units that combine gyroscopes and accelerometers to measure head angular velocities, as well as linear and angular accelerations (Table 1).

Head impact sensors vary in accuracy, with poor skull coupling leading to inaccurate impact counts and acceleration magnitudes. According to at least one report, skin-based and head-based sensors were displaced by up to 4 mm and 13 mm, respectively [45]. Helmeted systems are also limited in terms of the range of sports in which they can be employed, and skin-mounted sensors are prone to detachment during match-play, for example, due to the player sweating or during a tackle [21,23]. In contrast, mouthguards tightly coupled to the upper dentition exhibited less than 1 mm of displacement, making iMGs promising for precise measurement of head kinematics during contact events [45].

### 3.2. Practical Application

At the drill level, iMGs can help identify specific training drills linked to high-magnitude HAEs, providing valuable data for risk analysis to assess whether these drills are necessary from the perspectives of both performance and player welfare [46]. These devices can also be used to track HAE exposure for individual players, enabling the implementation of contact involvement management strategies to minimize unnecessary risk. Such data can further inform session design and the management of overall exposure, allowing drills associated with high-magnitude HAEs to be balanced with those resulting in lower-magnitude HAEs. Clinically, iMGs can alert medical professionals to high-magnitude HAEs that increase the risk of concussion, supporting more informed decision-making and aiding in the early detection of head impacts in collision sports [22]. Additionally, the head kinematic data captured by iMGs can improve understanding of brain injury mechanisms, offering insights that may support potential rule modifications in the interests of player welfare.

### 3.3. Limitations

Despite the potential benefits, several issues persist in relation to these new technologies and research in this area. Recent validation studies have raised concerns about validity, reliability, and clinical utility, citing error rates in filtering algorithms, a lack of video confirmation, low sensitivity in predicting concussive injury (sensitivity 17–96%, positive predictive value 0.08–0.33%), and a high prevalence of false negative HAEs (6–14%) [16,47,48,49]. Although video verification and post-processing algorithms provide effective solutions for reducing false positives, mitigating the occurrence of false negatives remains an issue for researchers [22]. Continuing to explore current limitations including the precision and reliability of the technology will enhance overall clinical confidence in the devices. Such an exploration may include considering whether classification algorithms need to be specifically designed and/or validated for each combination of sport, gender, and mouthguard system to ensure the clinical utility of valid and reliable data [24,47]. Another consideration regarding the broader application of these methods is that most contact events result in no recorded HAE or lower-magnitude HAEs [16]. Therefore, different thresholds and algorithms are likely to be needed to identify total numbers of contact events compared with contact events that result in an HAE. Consequently, it may be more appropriate for head impact telemetry devices such as iMGs to be used solely for the identification of HAEs rather than for quantifying all contact events in collision sports. Finally, the comfort of iMGs, particularly regarding their size and fit, has been identified as a significant barrier to its adoption by players. Additional challenges, including time constraints and a lack of understanding of the data, further limit meaningful engagement with the technology [50]. Addressing these concerns is essential to enhancing player and coach buy-in.

## 4. Video Analysis

### 4.1. Data Capture

Video analysis has long been the primary method for quantifying contact events in team sports, including rugby. While some critics have raised concerns about the potential for human error, citing this as a significant barrier to the widespread adoption of video analysis, many of these criticisms lack substantial supporting evidence [25,28]. In contrast, recent research has demonstrated that video analysis offers excellent intra-rater reliability, comparatively outperforming the sensitivity of tracking system-embedded MEMS-derived contact data [40]. With typical error rates below 8% in rugby sevens, intra- and inter-coder reliability levels with a CV of approximately 5%, and intraclass correlation coefficients ranging from 0.91 to 1.00 in rugby union, video analysis is widely regarded as the gold standard for capturing contact events in collision sports [17,25,40,51].

#### Practical Application

One of the main benefits of video analysis is its ability to differentiate between different contact events. Unlike other automated methods that have low sensitivity for distinguishing between unique contact events, video analysis allows visual detection of each specific event. Moreover, video analysis has been employed extensively in rugby union to assess tackling technique and its influence on successful contact, as well as risk of injury [52,53,54]. Video analysis has proved advantageous in providing a visual depiction of tackle events, allowing practitioners to better understand the context of successful and unsuccessful contact events. For example, head up and forward, counter-acting the ball-carriers fend, shoulder tackles targeted at the ball-carrier mid-torso, using the arms to wrap or pull, and leg driving are key tackler characteristics associated with positive tackle outcomes in rugby union [13,52]. Through video analysis, coaches can convey this information to athletes, facilitating the design of targeted training interventions aimed at enhancing these key performance attributes. This feedback loop, informed by visual evidence, contributes to a more focused and effective approach to skills development within the team. Unlike digital tracking technologies that require specialized equipment and expertise for their use, interpretation, and integration into team sports, video analysis is ubiquitous across all levels of sport and can be easily accessed and interpreted by a range of stakeholders, including coaches, data analysts, and players.

### 4.2. Limitations

It is important to acknowledge the drawbacks of video analysis in this context, particularly its labor-intensive nature that requires a video analyst to manually code each contact event. However, even with automated methods of data capture, analysts are likely to continue manually coding these contact events, as doing so provides additional insights for coaches that automated systems cannot currently offer. Some variables, such as latching and non-performance contact, demonstrate lower reliability, which is likely to be due to the novelty of coding these specific events [40]. Furthermore, variations in the definitions used to classify contact events have contributed to inconsistencies in reported values. For example, some studies have excluded contact events such as rucks and mauls when reporting contact frequency, though the rationale for this omission remains unclear [36].

To minimize errors in reporting, it is crucial that all contact events are accounted for at data collection and that the operational definitions used for video coding are clearly understood and consistently applied by all members of the multidisciplinary team. For example, performance analysts may consistently choose not to code late tackles or off-the-ball collisions, as these events often occur after play has stopped or away from the main passage of play and including such incidents could artificially inflate a player’s tackle count. However, from a practitioner’s perspective, these contacts can still be relevant, particularly when assessing the accumulation of tissue damage over time [40]. Ignoring these events could lead to important factors related to player welfare and injury risk being overlooked, thereby creating a gap between the captured data and the actual physical demands experienced by the athletes. Furthermore, capturing contact involvement through video analysis requires clear visual representation of each contact event, which can often be hindered by limited camera angles. This limitation is particularly evident in domestic or semi-professional competitions, where budget constraints and insufficient resources prevent the use of multiple camera angles. This issue is further compounded during training sessions, where multiple drills are conducted simultaneously across different areas of the field, increasing the likelihood of contact events being missed.

### 4.3. Data Integration

A more effective approach to quantifying contacts may involve the integration of video analysis and microtechnology [17,18]. By combining these methods, coaches, practitioners, and researchers can cross-reference microtechnology-derived data against video footage, improving data accuracy and yielding clearer differentiation of collision events. For this process to be seamless, effective communication between sport scientists and data analysts is essential, ensuring smooth workflows in data capture, analysis, and reporting to coaches. To accomplish this requirement, manufacturers should prioritize software integration rather than limiting data uploads to proprietary platforms. For example, Catapult currently blocks the integration of its MEMS-derived contact data into other video analysis software, such as e.g., SportsCode, confining its use to Catapult Vision. This restriction hinders the practicality of adopting a comprehensive workflow and reduces the overall utility of combining different technologies.

**Figure 1:**
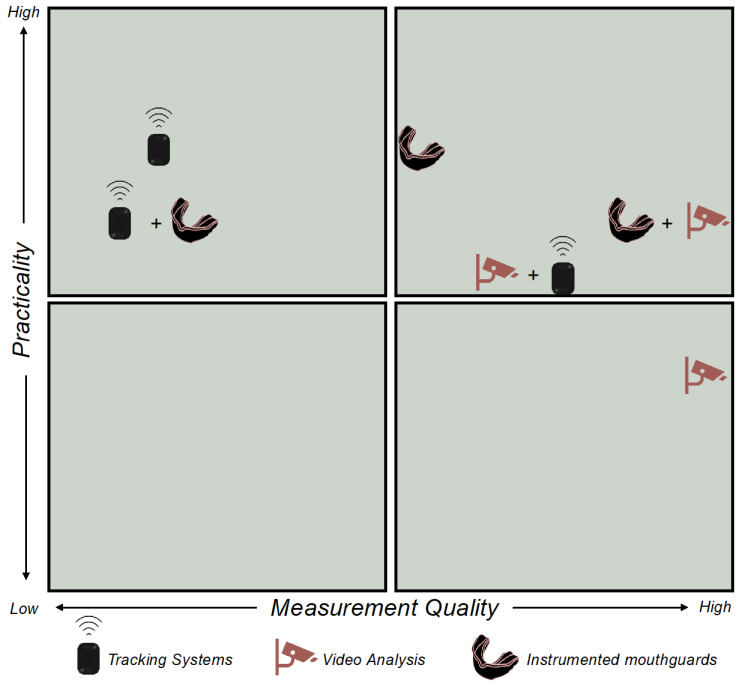
Quadrant illustrating the overall utility of individual and combined data capture methods based on subjective evaluation of measurement quality and practicality.

Figure 1 illustrates the overall utility of individual and combined methods of data capture. The utility rating for each method was based on subjective evaluations of both measurement quality and practicality. Measurement quality was assessed across three sub-variables: validity, consistency, and type of contact event, captured across various rugby codes and genders. Validity and consistency were rated as low, moderate, or high. The types of contact events captured were categorized as “All Impacts above a set threshold”, “HAE only”, or “All Contact”. Practicality was evaluated based on four sub-variables: cost, comfort, ease of data capture/use (each rated low, moderate, or high), and the ability to capture contact intensity/volume (yes/no). Each characteristic was assigned an integer score between 1 and 3, except for the contact intensity/volume measure, which received a value of 0 (no) or 3 (yes). Scores were subsequently summed and then divided by the total number of variables to generate x and y values ranging from 1 to 3. These values were subsequently plotted on a quadrant to visualize the overall utility of each method.

## 5. An Alternative to Quantifying Contact Intensity and Contact Volume

A primary limitation of video analysis is its ability to measure only the frequency of contact exposures without offering insights into the intensity of individual contact events (quantified according to specific levels of muscle activity in terms of rate of energy expenditure, force, or velocity) or the cumulative contact volume (the combined product of intensity, duration, and frequency of contact). Both of these are critical measures for providing greater insight into sporting demands, monitoring processes, and subsequent recovery timelines. There is a clear need to develop an effective and accurate means to capture such information.

The adoption of instrumented mouthguard technology or MEMS housed in tracking devices offers a promising alternative to video assessment for measuring contact events. However, since iMGs are specifically designed to measure head acceleration, they may not detect all contact events, particularly those not directly impacting the head. Furthermore, tracking system-embedded MEMS-derived contact intensity and volume metrics show substantial variability between studies, complicating researchers’ attempts to obtain a detailed understanding of the issues involved. Additionally, the intensity of contact events detected with this technology is difficult to compare longitudinally, given that the technology is constantly evolving and the numeric bins that group contact events based on their intensity differ between manufacturers. Although recent advancements in technology such as improved algorithms and MEMSs with higher specificity and sensitivity can provide better detection capabilities, there is still a lack of comprehensive studies to confirm these improvements.

An alternative approach to assessing contact intensity and volume might involve subjectively rating each contact event. Recently, subjective descriptions of contact intensity have been used to categorize individual contact events into different categories of intensity. This method assigns each contact to one of three categories—minimal, hard, or maximal, using weighted values of 1, 3, and 5, respectively—based on predefined criteria, and assessment is performed via video analysis [55]. This approach could be valuable for capturing static contact that might otherwise go undetected. Certain contact events, such as rucks in rugby union and rugby sevens, present unique challenges relating to data capture. A ruck is formed when the ball is on the ground and one or more players from each team who are on their feet close around it, competing for the ball. Given the static nature of rucks, these events are often harder to capture with microtechnology than dynamic actions like standing tackles. Similarly, when a player jumps to contest possession at a lineout and then lands in a maul, the postural deviation detected via microtechnology may be insufficient to classify this as a contact event. In such cases, subjective intensity ratings may provide valuable insights. Moreover, not all contact events are of equal significance. Qualitative descriptors that differentiate between minimal, hard, and maximal efforts can aid analysts in more accurately describing contact intensity. For example, if the defending team does not contest a ruck, resulting in minimal contact, the event may have limited physiological relevance and would therefore be categorized as involving minimal contact. Additionally, involvement in an attacking ruck where players bind to extend the ruck and protect the scrum-half during a box kick may involve minimal contact, thus posing limited risk of tissue damage. This approach gives performance staff and analysts the ability to generate an overall contact volume metric which accounts for both the frequency and intensity of all contact involvements [55].

**Figure 2:**
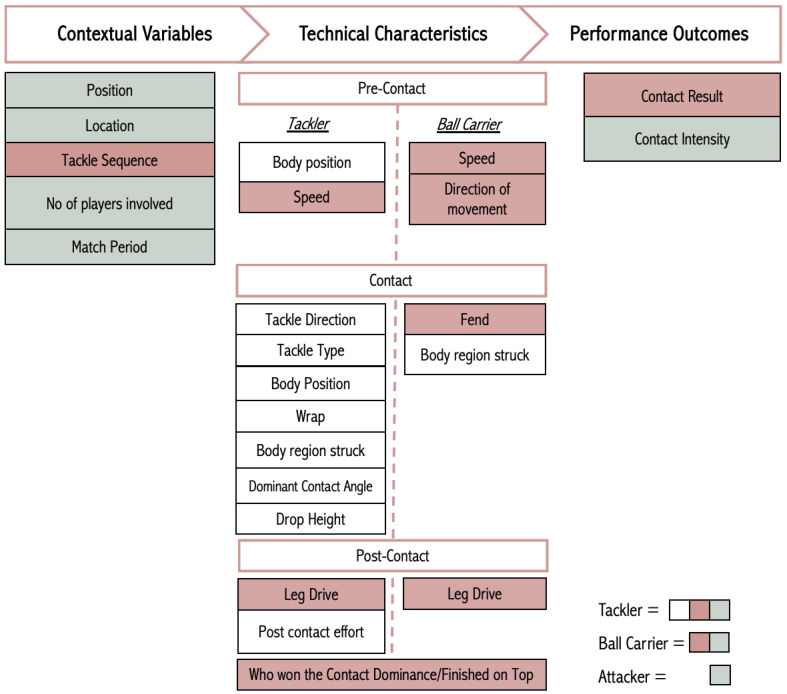
Depiction of the key variables for tacklers and ball-carriers adapted from the RUVAC video analysis framework [56], with the addition of attacker/latch. Each variable is accompanied by a set of descriptors used for modeling the subjective contact intensity rating assigned to each individual contact event. The colours correspond to the labelled variables associated with the coding of different type of contact events.

**Figure 3:**
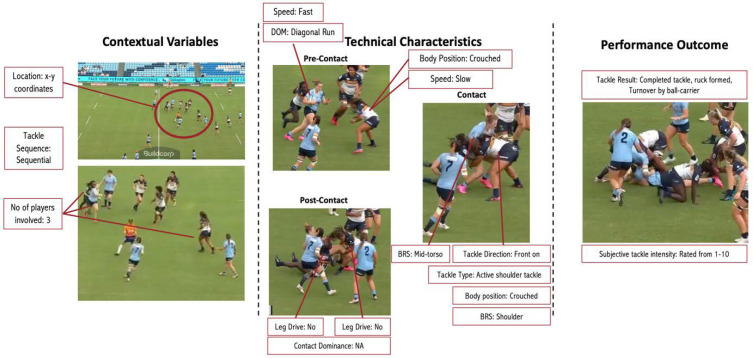
Example illustrating how a tackle event is notated during video analysis. This example involves a single ball-carrier and two tacklers. BRS: body region struck.

While this subjective categorical rating method shows promise, there is potential for improving its reliability. It may be possible to develop a predictive model utilizing common contact descriptors (Figure 2), exploring the relationship between subjective intensity ratings assigned by video analysts and these descriptors [56] (Figure 3). Using a series of machine learning algorithms to predict the intensity of a contact event (response variable) based on the contact descriptors (features) [56], the best performing features and machine learning algorithms could be determined and then applied to a test set to evaluate the model’s effectiveness on new unseen data. Such a model would reduce subjectivity and provide a deeper understanding of the external contact demands on players, offering valuable insights for tailoring training and recovery strategies to enhance performance.

In addition, biomechanical simulation and modelling approaches could be developed to quantify joint kinematics and internal forces involved in complex sporting movements such as sprinting or contact events in collision sports. For instance, subject-specific musculoskeletal models have been used to estimate internal forces on the cervical spine during high-impact rugby scrummaging [57]. Additionally, a musculoskeletal modelling and simulation framework designed for the analysis of sprinting biomechanics has enabled three-dimensional data-tracking simulations to replicate experimental kinematics and kinetics across different phases of sprinting [58]. By refining these methods, computational biomechanics may provide the means to enhance performance analysis, improve quantification of contact-related events, and strengthen injury-prevention strategies in collision sports.

## 6. Conclusions

Advancing the understanding of contact intensity and volume in rugby union requires a multifaceted approach. While traditional video analysis offers insights into contact frequency, it falls short of capturing the full spectrum of contact involvement (contact intensity and cumulative contact volume). Combining advanced technologies including instrumented mouthguards and microtechnology presents a promising solution. However, it may take some time before such technology provides reliable insights that can be used to inform decision-making. A new approach that integrates subjective ratings, such as those provided by analysts, with commonly used descriptors could leverage predictive modelling and machine learning to enhance the accuracy and depth of contact assessment. Development and validation of new models will provide valuable insights into the contact demands of collision sports and inform more effective training and injury-prevention strategies.

## Figures and Tables

**Table 1:** Overview of micro sensors and their application in contact event detection.

Sensor	Function	Application
Accelerometer	Measures linear acceleration in three dimensions (x, y, and z axes). In sports, an accelerometer captures changes in velocity to obtain linear movement data.	These sensors detect changes in velocity which may be due to gravity (e.g., jumping, falling) or the athlete’s own motion (e.g., sprinting, decelerating, changing direction).
Gyroscope	Measures rotational motion or angular velocity (rate of rotation) around the x, y, and z axes. Gyroscopes help detect changes in orientation, providing rotational motion data.	The ecological validity of tackle detection depends on the sensor type, algorithm, and the sport analyzed. An acceleration threshold alone may not be able to separate contact from impact, whereas assessing body lean with a gyroscope in combination with the use of acceleration thresholds can improve the ecological validity.
Magnetometer	Measures the strength and direction of magnetic fields. Magnetometers are often used to determine orientation relative to the Earth’s magnetic field (i.e., compass direction).	The magnetometer senses the surrounding magnetic field; combined with the accelerometer and gyroscope data it provides accurate information about the test subject’s orientation in space, further improving contact detection.

**Table 2:** Advantages and disadvantages of individual and combined methods of data capture.

Methodology	Advantages	Disadvantages
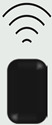	Time-efficient, with automated data capture; No additional demands on athletes or staff; No additional cost; Provides volume- (frequency × intensity) and intensity-derived metrics.	Potential issues with validity and reliability/poor sensitivity in identifying contact events; Each device/update requires independent verification of its validity and reliability; New metrics lack clear explanations of their derivation; Variability in algorithms and filtering methods across manufacturers complicates comparative analysis; Variability in vest sizes and clothing affects data accuracy.
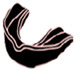	Automated data capture; Provides volume- (frequency × intensity) and intensity-derived metrics; Identifies specific training drills associated with high-magnitude HAEs; Early detection of head impacts, improving player welfare; Enhances understanding of brain injury mechanisms.	High prevalence of false positives and false negatives; Low specificity in predicting concussive injuries; Increased cost; Different thresholds and algorithms may be required in order to quantify total contact events. Increased effort needed to incorporate into existing workflows. Limited data integration with other technology.
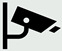	High sensitivity in distinguishing between different types of contact (e.g., tackle vs. ruck); Allows assessment of tackle technique and its impact on contact success; Provides visual feedback, aiding coach evaluations and skills development.	Labour-intensive process. Limited camera angles can reduce accuracy. Operational definitions used in coding must be clearly understood by analysts. Inconsistent coding of contact events. No volume or intensity-derived metrics are available.
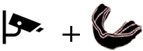	Assists with syncing iMG data at the drill level by integrating drills with tracking system software and iMG-derived HAE data (not currently available).	Limited video verification of contact events. Additional time and effort required for data capture, cleaning, and reporting.
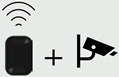	Improved sensitivity and reliability when combined.	Restricted integration between manufacturers. Labour-intensive process when integrating video analysis. Requires video verification during training, limiting its practicality and reducing automation in data capture.
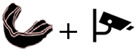	Improved sensitivity and reliability when combined; Improved detection of head impacts.	Labour-intensive process when integrating video analysis. Requires video verification during training, limiting its practicality and reducing automation in data capture.

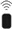
: tracking systems; 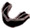: instrumented mouthguard; 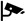: video analysis; HAEs: head acceleration events, iMG: instrumented mouthguard.

## Data Availability

No new data were created or analyzed in this study. Data sharing is not applicable to this article.
